# Age-Stratified Analysis of Vaginal Microbiota Dysbiosis and the Relationship with HPV Viral Load in HPV-Positive Women

**DOI:** 10.1155/2022/1372926

**Published:** 2022-07-27

**Authors:** Mingzhu Li, Chao Zhao, Yun Zhao, Jingran Li, Lihui Wei

**Affiliations:** Department of Obstetrics and Gynecology, Peking University People's Hospital, No. 11 Xizhimen South St, Beijing 100044, China

## Abstract

**Objective:**

This study evaluated the distribution of vaginal microbiota dysbiosis and the association with HPV viral load test in high-risk HPV-positive women before and after 50 years old.

**Methods:**

For this cross-sectional study, 388 HPV-positive women prior to referral to colposcopy in Peking University Peoples' Hospital were included and classified as younger than 50 years (*n* = 307) and aged 50 years or older (*n* = 81), midvagina bacterial community composition was characterized by FlashDetect™ MAX vaginal microbe detection kit, and BMRT-HPV reported type-specific viral loads/10,000 cells.

**Results:**

The community state type (CST) IV was the most common CST occurring in 148 women (38.1%). The proportion of CST IV in those aged 50 years or older was significantly higher than those younger than 50 years (women) (66.7% vs. 30.6%); the difference was statistically significant (<0.001). CST distribution has no statistical difference in different grades of cervical lesion, regardless of the age (*p* = 0.238 and 0.263). However, the women with high-grade cervical lesion presented a more complicated trend and the abundance of vaginal microbiota dysbiosis than low-grade lesion. HPV16/18 viral load was found to be significantly higher in CST III and CST IV than CST I/II/V (*p* < 0.05)in women younger than 50 years.

**Conclusions:**

In women younger than 50 years, higher HPV16/18 load was more closely associated with CST IV; however, it had no significant correlation in women aged 50 years or older.

## 1. Introduction

Persistent infection with high-risk human papillomavirus (hrHPV) is the main cause for the development of cervical intraepithelial neoplasia (CIN) and cervical cancer [1, 2]. Despite the controversy of the association between high viral load and the severity of cervical lesions [3, 4], it has been reported recently that HPV viral load increased with the grade of cervical lesions and is as sensitive as Cobas4800 for primary cervical cancer screening [5].

It is still unclear why some hrHPV infections resolve clinically while others persist and cause dysplasia and even cervical cancer. Many factors such as age, smoking, HIV infection, and oral contraceptives are associated with persistent HPV infection [6]. In addition to these variables, it has been proposed that the vaginal microbiota plays an important role in the development of HPV infection leading to cervical neoplasm [7]. Some vaginal microorganisms, such as *Gardnerella*, *Atopobium*, *Enterococcus*, *Streptococcus*, *Fusobacteria*, and *Mycoplasma*, as well as a decrease in the proportion of *Lactobacillus* spp., have been linked to dysbiosis that would generate an unstable microenvironment, which in turn could enable the effect of key risk factors in cervical cancer [8, 9]. However, it has been reported with greater evidence that the postmenopausal women had lower abundance of Lactobacillus species, and a more diverse community of vaginal microbes, which attributed to decreased estrogen, reduced glycogen content in vaginal epithelial cells and limits the energy source for lactobacilli [10].

To determine whether the composition of vaginal microbiota and HPV load differs before and after 50 years old, we conducted a pilot analysis of the association between vaginal microbiota, HPV viral load, and the risk of high-grade cervical lesion in different aged HPV-positive women.

## 2. Materials and Methods

### 2.1. Study Design

A total of 388 women with hrHPV positive results prior to referral to colposcopy in Peking University People's Hospital were invited to participate in this study from Nov. 2020 to Nov. 2021. The study was approved by the Ethics Committee of Peking University People's University (No. 2020PHB298-01), and written informed consent was obtained from all participants after providing detailed information about the study and its characteristics. These women were divided into the <50-year age group (*n* = 307) and ≥50-year-old group (*n* = 81), according to the previous report, which has been showed that the threshold of 50 years distinguished the peaks associated with very low lactobacilli-dominated microbiota community type and bacterial compositions dominate the cervicovaginal microbiota in women aged 50 years or older [11]. Participants who had acute vaginitis or cervicitis and vaginal antimicrobials or received estrogen replacement therapy, had sexual intercourse within 72 hours, are pregnant or up to 2 months postpartum, had a history of hysterectomy, or had immune system disease were excluded from the study. The sample collection was completed before colposcopy procedure, a vaginal speculum was placed to expose the cervix, then cervical exfoliated cell sample at the squamocolumnar junction of the cervix was obtained with a sampling brush, and then the brush was placed into a 20 mL PreservCyt1 solution (Hologic, Marlborough Mass, USA) for testing. The samples were stored at 4°C within 72 hours and at -20°C for long-term preservation. All samples were tested with cytology first, and the remaining preservation solution was used for HPV genotype, viral load, and vaginal microbes, respectively. Pathologic confirmation was performed by colposcopy-directed punch biopsy for all participants. Cervical biopsies under colposcopy were then histologically examined and classified according to the 2014 World Health Organization (WHO) Classification of Tumors of the Female Genital Tract, namely, low-grade squamous intraepithelial lesion (LSIL/CIN1) and high-grade squamous intraepithelial lesion (HSIL/CIN2 and HSIL/CIN3) [12].

### 2.2. BMRT HPV PCR Assay

The BMRT is detected based on PCR-based high-risk HPV assay, which was performed with the fluorescence-based multiplex HPV DNA genotyping kit (Bioperfectus Ltd., Jiangsu, P.R. China). PCR primers and corresponding TaqMan probes were developed for the 21 most prevalent HPV types to amplify the HPV L1 gene, including 14 hrHPV genotype (HPV16, 18, 31, 33, 35, 39, 45, 51, 52, 56, 58, 59, 66, and 68) and 7 medium-risk and low-risk HPV genotypes (HPV26, 53, 82, 73, 6, 11, and 81). For this study, only hrHPV was used in the statistical analysis of this study. A single-copy gene encoding DNA topoisomerase III (human TOP3) was amplified in the reaction to control DNA quality and determine the relative viral copy numbers in the samples. The normalization of HPV type-specific viral loads was performed as follows: viral load = log10[Cn HPV/Cn TOP3 × 10,000] copies/10,000 cells, where Cn HPV is the quantity of HPV DNA and Cn TOP3 is the number of human cells. The experimental procedure was conducted according to the kit manufacturer's instructions, the detailed process was described by Dong et al. and Duan et al. [5, 13].

### 2.3. Vaginal Microbes Detect

Nucleic acid of vaginal microbes was extracted from 0.5 mL of vaginal swab samples using a modified TIANamp Virus DNA/RNA kit (Tiangen, China). Samples were lysed and homogenized twice with 0.2 mL lysis buffer GA and 0.3 g zirconia beads for 2 minutes by Biospec MiniBeadbeater-16 in 2 mL tube. The supernatant was treated with 40 mL protease K and 0.4 mL carrier RNA working solution at 56°C for 15 min in a 1.5 mL tube. 500 mL of ethanol was then added and mixed for 15 sec and placed for 5 min at room temperature. Sample solution was transferred into RNase-free column CR2 set and centrifuged 6,000 g for 1 min. The column CR2 was washed with 0.5 mL of buffer GD, 0.6 mL of buffer PW twice, and 500 mL of ethanol by centrifuging 6,000 g for 1 minute in turn. The column CR2 was dry and centrifuged 13,400 g for 3 min. The nucleic acid was eluted with 50 mL RNase-free ddH2O.

Vaginal microbes were detected using FlashDetect MAX vaginal microbe detection kit (Coyote, Beijing, China) in which detection and quantitation of target sequence from vaginal microbes are associated with bacterial vaginosis, aerobic vaginitis, vulvovaginal candidiasis, trichomoniasis, chlamydia, mycoplasma, sexually transmitted infection, including *Lactobacillus* (*L. gasseri*, *L. crispatus*, *L. jensenii*, and *L. iners*), *Gardnerella vaginalis*, *Atopobium vaginae*, *Bacterial vaginosis-associated bacteria 1/2* (*BVAB1/2*), *Bacterial vaginosis-associated bacteria TM7* (*BVAB-TM7*), *Megasphaera spp.*, *Prevotella fragilis*, *Mobiluncus*, *Sneathia amnii*, *Candida* (*C. spp.*, *C. krusei*, and *C. glabrata*), *Escherichia coli*, *Enterococcus faecalis*, *Staphylococcus aureus*, *Streptococcus pyogenes*, *Streptococcus agalactiae*, *Ureaplasma urealyticum*, *Mycoplasma hominis*, *Mycoplasma genitalium*, *Chlamydia trachomatis*, *Trichomonas vaginalis*, *Treponema pallidum*, *Neisseria gonorrhoeae*, *Haemophilus ducreyi*, *Herpes simplex 1*, and *Herpes simplex 2*. Real-time PCR was performed in a total volume of 20 *μ*L (2 *μ*L of extracted nucleic acid samples, 8 *μ*L of VM reaction mix, and 10 *μ*L of VM enzyme mix) in a 96-well plate using a Roche 480 (Roche Applied Sciences, Mannheim, Germany). Human RNase P gene and 16S gene were used as the internal reference gene. Reactions were incubated at 95°C for 2 min, followed by 40 cycles of denaturation at 94°C for 5 seconds and annealing and extension at 60°C for 30 seconds. The detection curve of sample has a significant exponential amplification curve with the Ct value ≤ 33; the detection result is positive.

### 2.4. Statistical Analysis

Statistical analysis was performed using SPSS 26.0 (IBM Corp., Armonk, NY, USA). Categorical variables were presented as frequencies and percentage (histopathological results, cervical cytology, hrHPV genotype, etc.). Viral load was measured as RLU/PC ratios (RLUs) and agreed with previous specifications in the hybrid capture assay. Viral quantification data in RLUs were initially continuous measurements. RLUs were transformed into their logarithm (Log10). Quantitative data were described by the mean ± standard deviation (age). Nonnormally distributed continuous variables presented as median and interquartile range (IQR) (all HPV viral load, HPV16/18 viral load, and hr16/18 viral load (Log10)). Differences in frequencies for categorical variables between cases and controls were evaluated using the chi-square statistic with Yates correction. The *p* values for community state types (CSTs), microbiome composition, and cervical lesion were calculated using a *χ*^2^ test. Abundance patterns within each age group and each microbiota community type were clustered by a hierarchical clustering algorithm via Ward's method. The patterns were scaled column-wise. The species selected for the heatmaps correspond to five types of CST.

## 3. Results

### 3.1. Characteristics of the Study Population

A total of 388 women were enrolled into the study and classified into <50-year group (*n* = 307) and ≥50-year group (81). The characteristics of each group are shown in Table 1. The media age was 41.5 (±10.7) (range: 19-77). 93.8% of women aged ≥50 years and 1.9% of women aged <50 years were postmenopausal; the difference was statistically significant. There were no significant differences in age, cytology, HPV status, HPV load, and the degree of cervical lesion between the two groups (*p* > 0.05). About the CST distribution, CST IV was the most common type occurring in 148 women (38.1%), CST III accounted for 35.6% and took the second place. While CST II and V accounted for the least (1.3% and 2.6%). The proportion of CST I and III in those younger than 50 years was significantly higher than those aged 50 years or older women. However, the proportion of CSTIV in those aged 50 years or older was significantly higher than those younger than 50 years women (66.7% vs. 30.6%); the difference was statistically significant (<0.001).

### 3.2. Characteristics of Vaginal Community State Types (CSTs) for Different Groups

**Figure 1:**
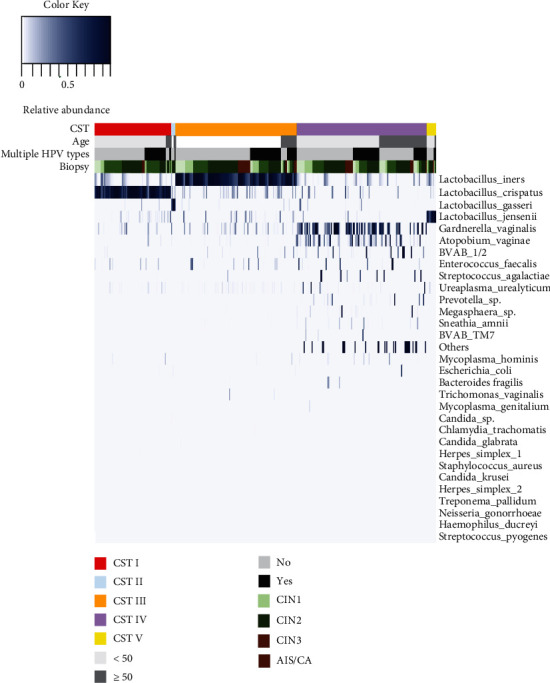
Vaginal microbiome composition according to age, HPV status, cervical lesion, and vaginal community state types.

Hierarchical clustering of the cervical microbiota based on the type and relative abundances of the bacterial taxa revealed that all samples clustered into five major groups: CST I, CST II, CST III, CST IV, and CST V (Figure 1). CST I was dominated by L crispatus and found in 87 women (22.4%). CST II was dominated by *L. gasseri* and present in only five women (1.3%). CST III and CST V were dominated by *L. iners* (35.6%) and *L. jensenii* (2.6%), respectively. CST IV was characterized by a diverse and complex array of facultative and strictly anaerobic BV-associated bacteria (*Gardnerella*, *Atopobium*, *BVAB_1/2*, *Enterococcus*, *Streptococcus*, *Ureaplasma*, *Prevotella*, Megasphaera, *Sneathia*, et al. and very low numbers of *Lactobacillus*) and mostly dominated in the older than 50 age group. The proportions of CSTs in different stage of cervical lesion are shown in Table 2. Although the proportions of CST IV were gradually augmented with the progression of the severity of CINs (CIN3 : 40.4% > CIN2 : 23.4% > CIN1 : 13.8% > normal : 12.8%) in women younger than 50 years, but there was a significant decrease in cervical cancer and adenocarcinoma in situ (AIS); there has no significant difference in both age group (*p* = 0.238 and 0.263). In women older than 50 years, CST I, CST II, and CST V decreased significantly, while CST III and CST IV were dominant in cervical cancer and AIS.

### 3.3. Identification and Cluster Analysis of Vaginal Microbiota in Different Group

**Figure 2:**
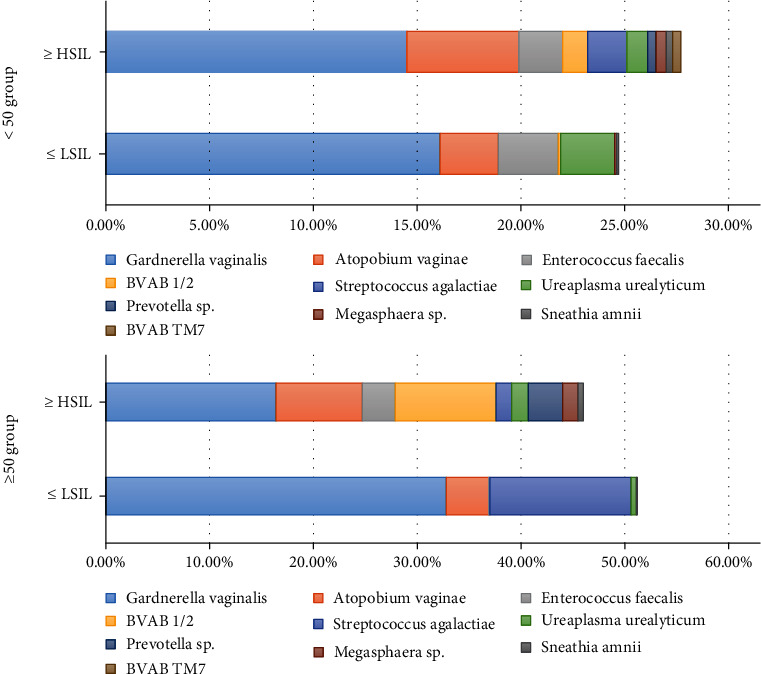
Average relative abundances of the 10 prevalent genera in vaginal microbiota in different degree of cervical lesion of different age. Note: ≥HSIL including histopathologically confirmed HSIL/CIN2, HSIL/CIN3, SCC, and AIS; ≤LSIL including histopathologically confirmed LSIL/CIN1 and normal.

As Figure 2 shown, in the CST of patients with histopathologically confirmed ≤LSIL group, the predominant type of bacteria was Gardnerella vaginalis. The structure of cervicovaginal microbiota of ≤LSIL individuals is relatively single. Gardnerella vaginalis occupy the main composition with 16.1% vs. 32.8% in <50-year- and ≥50-year-old women, respectively. However, the proportion of *Gardnerella vaginalis* was gradually reduced in histopathologically confirmed ≥HSIL group, compared with ≤LSIL individuals (≥HSIL: 14.5% vs. ≤16.1% in<50-year-old women; ≥HSIL:16.4% vs. ≤32.8% in ≥50-year-old women). Besides, the women with HSIL presented a complicated trend and the abundance of *Gardnerella vaginalis* (14.5%), *Atopobium vaginae* (5.4%), and *Enterococcus faecalis* (2.1%) was the predominant bacteria type in <50-year-old women. ≥50-year-old women diagnosed with HSIL exhibited abundant *Gardnerella vaginalis* (16.4%), *BVAB 1/2*(9.7%), and *Atopobium vaginae* (8.3%).

### 3.4. The Relationship of CSTs and HPV Viral Load

**Figure 3:**
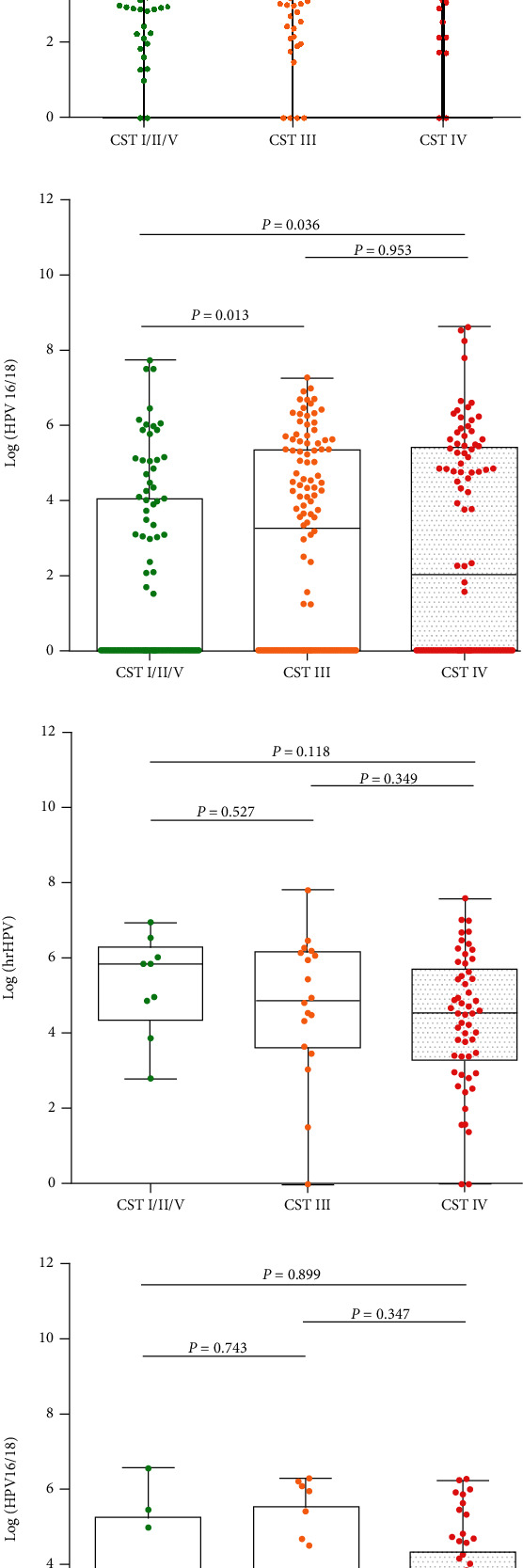
HPV viral load for different vaginal CSTs. (a) hrHPV load < 50 years old; (b) HPV16/18 load < 50 years old; (c) hrHPV load ≥ 50 years old; (d) HPV16/18 load ≥ 50 years old.

In women younger than 50-year-old group, there was no statistical significance between the CST type and all HPV viral load (*p* > 0.05). HPV16/18 viral load was found to be significantly higher in CST III and CST IV than CST I/II/V (*p* < 0.05). However, in women aged 50-year-or-older group, there was no significant difference in HPV viral load regardless genotype among CST types, as shown in Figure 3.

## 4. Discussion

HPV persistent infection leads to changes in the cervical microenvironment. It has been reported that the risk of high-grade CIN was dependent on both the HPV genotype and viral load, especially for HPV16 [2]. Besides, many studies suggest that the cervicovaginal microbiota changes are closely related to HPV infection, persistence of infection, and HSIL [14], but the relationship with the HPV viral load was unclear. Menopausal women were more likely to develop HPV persistent infection; the possible reason was the decreased estrogen levels after menopause, leading to changes in cervicovaginal microbiota, which lost its protection against pathogens including HPV [15].

Community state type (CST) was introduced first by Ravel in 2011 [16], which was used to describe the vaginal microbiota status and was divided into five groups: CST I, CST II, and CST V were normal vaginal microbiota, dominated by *Lactobacillus crispatus*, *Lactobacillus gasseri*, and *Lactobacillus jensenii*, respectively. CST III type, dominated by *Lactobacillus iners*, represented the subhealth state of vaginal microbiota, which was most likely to transform to CST IV type, whereas the fifth CST IV has lower proportions of *Lactobacillus* spp. and higher proportions of anaerobic organisms including *Mobiluncus* spp. and *Atopobium vaginae*.

In this study, in all the HPV-infected women, cervicovaginal microbiota were dominated by CST IV (38.1%) and CST III (35.6%); just as Chen et al. [17] reported that the most dominant CST in the HPV positive groups (HPV, LSIL, HSIL, and cancer) was CST IV, while CST II and CST IV accounted for the least in Chinese HPV positive women. It has been reported that Lactobacillus dominated the cervicovaginal microbiota of health and HPV transient infection women, while the microbiota diversity of HPV persistent infection patients significantly increased and the proportion of anaerobic bacteria in the composition significantly increased [15]. Although the healthy women were not included in our study, we found CST IV was more dominant in women aged 50 years or older (66.7%) than in women younger than 50 years (30.6%) when stratified by different age thresholds. Although postmenopausal women were less likely to have a CST dominated by Lactobacillus spp. than premenopausal women, nearly 50% of postmenopausal women have a high relative abundance of Lactobacillus spp. [10], and the presence of Lactobacillus spp. has been associated with exogenous hormone use [18]. In our study, this rate was higher, which may be related to the fact that all the participants were HPV-positive and exogenous estrogens were excluded. Burton et al. [19] reported *Atopobium vaginae* as a common member of the vaginal microbiota of postmenopausal women, and Brotman et al. found a distinct bacterial community state (CST IV-A) with low relative abundance of *Lactobacillus* was associated with vulvovaginal atrophy [20]. After menopause, the composition of vaginal microbiota has been shown to be less likely dominated by Lactobacillus spp. and more likely to be composed predominantly of anaerobic and aerobic bacteria [20].

Besides, our result showed that a proportion of CST IV was gradually increased with the progression of CIN severity, but decreased in cervical cancer, although there was no significant difference, which is different from the Chen et al.'s findings that the proportions of CST IV were gradually augmented with the progression of the severity of CINs (cancer > HSIL > LSIL) [17]. However, CST III and CST IV, not CST I, II, and V, were dominant in cervical cancer and AIS, especially in women aged 50 years or older.

Besides, it has been reported that the vaginal microbiota differences were primarily attributed to HPV infection (or subtype) and not SILs, indicating that infection itself may lead to changes in the vaginal microbial community [21].

It has been reported that HPV infection increased vaginal bacterial richness and diversity regardless of the status of CINs [17]. About CIN, there was a research finding that *Pseudomonas stutzeri*, *Bacteroides fragilis*, *Lactobacillus delbrueckii*, *Atopobium vaginae*, and *Streptococcus agalactiae* were associated with CIN status [22]. Wu et al. found that *Delftia genus* might be a microbiological hallmark of cervical lesion [23] In our study, we found that the predominant type of bacteria younger than 50 years was Gardnerella vaginalis, *Atopobium vaginae*, and *Enterococcus faecalis* regardless of low-grade or high-grade CIN. Among the women aged 50 years or older, except the *Gardnerella vaginalis* and *Atopobium vaginae*, *Streptococcus agalactiae* and *BVAB* 1/2 were the predominant types in the ≤LSIL group and the ≥HSIL group, respectively, further suggesting that the vaginal microbiome of HPV-infected women was associated with different ages. We also found that the abundance of *Gardnerella vaginalis* was gradually reduced and bacterial diversity increased with the progression of CINs severity, which was consistent with the previous report [17, 24]. However, the ≥HSIL group presented a complicated trend and the abundance of vaginal microbiota diversity than the ≤LSIL group; this is different from some other studies reporting that there was no association between the diversity of vaginal microbiome and the CIN progression [25, 26]. Over all, in our study, *Gardnerella vaginalis* and *Atopobium vaginae* dominated the cervicovaginal microbiota in both LSIL and HSIL+ women, which were thought to be associated with bacterial vaginosis (BV) [27] and had a higher CIN risk [28].

Besides, one of the key findings of our study was that HPV16/18 viral load was found to be significantly higher in CST III and CST IV in women younger than 50 years old; however, there was no connection between CST type and HPV viral load in women older than 50 years old. An association between hrHPV viral load in cervical samples and severity of prevalent cervical disease was first described in 1999 [29] and replicated in numerous studies [30–32] and proved that HPV16/18 was more sensitive and specific than 12 other subtypes of hrHPV, suggesting that HPV viral load is a type-specific biological indicator of cervical cancer [33]. It has been approved that a microbial environment with a higher proportion of anaerobic bacteria and a lower proportion of *Lactobacillus* spp. is more likely to HPV infection, and CST IV was related with an increased risk of transitioning to an HPV positive state [34, 35]. Besides, some studies identified cervicovaginal microbiota dysbiosis closely related to HPV persistent infection, and transition between clusters was more frequent in women with persistent HPV16 infection (34%) than in women with transient infection (19%) [15, 36] Based on the relationship with high HPV16/18 viral load in our study, CST IV has an enhanced effect on the risk of cervical high-grade lesions in women younger than 50 years old. However, for women over 50 years of age, vaginal microbiota dysbiosis is more common regardless normal or cervical lesions, as evidenced by the high proportion of CST IV in this study, so there is no significant association with HPV viral load among this population.

The strength of this study is that it is stratified by age to interpret the vaginal microbial compositions of HPV-positive women with different stages of cervical lesion and the relationship with HPV viral load, which has not yet been well elucidated. The limitations of this study were that it was a cross-sectional study. Hence, we could not conclude any causal relationship between the vaginal microbiome and HPV viral load or CIN diseases. We have to conduct longitudinal studies to study relationships between the dynamics of the vaginal microbiome and the HPV viral load and the progression or remission of CIN diseases. In addition, the underlying biological mechanisms also need to be detailed. Finally, we lack information about covariates that could affect the vaginal microbiota, such as status of smoking, menstrual cycle, sexual behavior, and hormonal contraceptives use [37, 38]. However, previous researches have shown small or no significant effects of the menstrual cycle stage or of use of hormonal contraceptives on the vaginal microbiota [39, 40]. Sexual behavior has also been shown to affect the vaginal microbiota [41]; therefore, we try to avoid the influence of this factor on population enrollment in this study.

## 5. Conclusion

By analyzing the vaginal microbial distribution of HPV infected people of different ages triaged by 50 years, our study found that women aged 50 years or older had higher type IV distribution and had no significant correlation with HPV load; However, higher HPV16/18 load was more closely associated with CST IV in women younger than 50 years.

## Figures and Tables

**Table 1:** Characteristics of 388 HPV-positive women in different age distribution (*n* (%)).

	<50 *n* = 307	≥50 *n* = 81	*p* value
Age			
*n*, mean ± SD	41.5 (±10.7)		
Menopause status	6 (1.9)	76 (93.8)	<0.001
Biopsy			
Normal	34 (11.1)	14 (17.3)	0.452
LSIL/CIN1	38 (12.4)	9 (11.1)	
HSIL/CIN2	86 (28.0)	19 (23.5)	
HSIL/CIN3	122 (39.7)	29 (35.8)	
SCC/AIS	27 (8.8)	10 (12.3)	
Cytology			
NILM	160 (52.1)	42 (51.9)	0.849
ASCUS/LSIL	69 (22.5)	14 (17.3)	
ASC-H+	78 (25.4)	25 (30.7)	
CST type			
I	81 (26.4)	6 (7.4)	<0.001
II	3 (1.0)	2 (2.5)	
III	120 (39.1)	18 (22.2)	
IV	94 (30.6)	54 (66.7)	
V	9 (2.9)	1 (1.2)	
HPV viral load			
Log (all); *n*, median (IQR)	4.79 (3.79-5.68)	4.60 (3.37-5.75)	0.436
Log (16/18); *n*, median (IQR)	1.44 (0-4.82)	1.52 (0-4.44)	0.556
Log (hrHPV); *n*, median (IQR)	4.78 (3.68-5.66)	4.52 (3.30-5.72)	0.365
Multiple HPV types			
Yes	90 (29.3)	30 (37.0)	0.181
No	217 (70.7)	51 (63.0)	
HR-HPV			
HPV16/18(+)	142 (47.5)	36 (46.2)	0.833
Other HR(+)	157 (52.5)	42 (53.8)	

LSIL: low-grade squamous intraepithelial lesion; HSIL: high-grade squamous intraepithelial lesion; CIN: cervical intraepithelial neoplasia; SCC: squamous cell carcinoma; AIS: adenocarcinoma in situ; NILM: negative for intraepithelial lesions or malignancy; ASCUS: atypical squamous cells of undetermined significance ASC-H: atypical squamous cell-cannot exclude HSIL; CST: community state types.

**Table 2:** The proportions of CSTs in different stage of histopathologically confirmed cervical lesions (*n* (%)).

	Normal	LSIL/CIN1	HSIL/CIN2	HSIL/CIN3	SCC/AIS	*p* value
Age < 50						
CST I, CST II, CST V	8 (8.6)	9 (9.7)	36 (38.7)	36 (38.7)	4 (4.3)	0.238
CST III	14 (11.7)	16 (13.3)	28 (23.3)	48 (40.0)	14 (11.7)	
CST IV	12 (12.8)	13 (13.8)	22 (23.4)	38 (40.4)	9 (9.6)	
Age ≥ 50						
CST I, CST II, CST V	1 (11.1)	1 (11.1)	3 (33.3)	4 (44.4)	0 (0.0)	0.263
CST III	2 (11.1)	4 (22.2)	3 (16.7)	4 (22.2)	5 (27.8)	
CST IV	11 (20.4)	4 (7.4)	13 (24.1)	21 (38.9)	5 (9.3)	

CST: community state types; LSIL: low-grade squamous intraepithelial lesion; HSIL: high-grade squamous intraepithelial lesion; CIN: cervical intraepithelial neoplasia; SCC: squamous cell carcinoma; AIS: adenocarcinoma in situ.

## Data Availability

The datasets generated during and/or analyzed during the current study are available from the corresponding author on reasonable request.
